# Population-based studies of relationships between dietary acidity load, insulin resistance and incident diabetes in Danes

**DOI:** 10.1186/s12937-018-0395-1

**Published:** 2018-10-06

**Authors:** Joachim Gæde, Trine Nielsen, Mia L. Madsen, Ulla Toft, Torben Jørgensen, Kim Overvad, Anne Tjønneland, Torben Hansen, Kristine H. Allin, Oluf Pedersen

**Affiliations:** 1 The Novo Nordisk Foundation Center for Basic Metabolic Research, Section of Metabolic Genetics, Faculty of Health and Medical Sciences, University of Copenhagen, Blegdamsvej 3B, DK-2200 Copenhagen, Denmark; 2 Research Centre for Prevention and Health, Copenhagen, the Capital Region of Denmark Denmark; 3 Department of Public Health, Faculty of Health and Medical Sciences, University of Copenhagen, Copenhagen, Denmark; 4 Faculty of Medicine, Aalborg University, Aalborg, Denmark; 5 Danish Cancer Society Research Center, Copenhagen, Denmark; 6 Department of Clinical Epidemiology, Bispebjerg and Frederiksberg Hospital, Copenhagen, the Capital Region of Denmark Denmark

**Keywords:** Dietary acid load, PRAL, Glucose, Insulin resistance, Disposition index, Type 2 diabetes

## Abstract

**Background:**

It has been suggested that the acidity of the diet may be related to increased risk of type 2 diabetes. To investigate this hypothesis, we tested if the acidity of the diet, measured as the Potential Renal Acid Load (PRAL) score, was associated with incident diabetes and diabetes-related intermediary traits.

**Methods:**

A total of 54,651 individuals from the Danish Diet, Cancer and Health (DCH) cohort were included in the prospective cox regression analyses of incident diabetes over a 15 years follow-up period. Moreover, 5724 Danish individuals with baseline data from the Inter99 cohort were included in the cross sectional, multivariate and logistic regression analyses of measures of insulin sensitivity, insulin release and glucose tolerance status derived from an oral glucose tolerance test (OGTT).

**Results:**

In the DCH cohort a trend analysis showed that quintiles of PRAL score were, after multifactorial adjustment, associated with a higher incidence of diabetes (*p*_trend_ = 6 × 10^− 7^). HR for incident diabetes was 1.24 (1.14; 1.35) (*p* = 7 × 10^− 7^) between first and fifth PRAL score quintile.

In Inter99 higher PRAL score associated with insulin resistance as estimated by lower BIGTT-Si (an OGTT-derived index of insulin sensitivity) (*p* = 4 × 10^− 7^) and Matsuda index of insulin sensitivity (*p* = 2 × 10^− 5^) as well as higher HOMA-IR (*p* = 0.001). No association was observed for measures of insulin release, but higher PRAL score was associated with lower OGTT-based disposition index.

**Conclusions:**

A high dietary acidity load is associated with a higher risk of diabetes among middle-aged Danes. Although adjustment for BMI attenuated the effect sizes the association remained significant. The increased risk of diabetes may be related to our finding that a high dietary acidity load associates with impaired insulin sensitivity.

**Electronic supplementary material:**

The online version of this article (10.1186/s12937-018-0395-1) contains supplementary material, which is available to authorized users.

## Background

Accumulating evidence suggests that a high dietary acidity load results in chronic tissue metabolic acidosis which, in turn, may contribute to the development of insulin resistance and type 2 diabetes (T2D) [1–5].

Observational studies report that a high dietary acidity load associates with the risk of developing T2D: an epidemiological study of ~ 66,000 middle-aged French women, including 1372 incident T2D cases, reported a higher incidence of T2D during 14 years of follow-up in those study participants with a high dietary acidity load [6]. Also, in an analysis combining data from three observational studies of American health professionals with a total of 15,305 cases of T2D in 4,025,131 person years of follow-up, the authors reported an increased risk of T2D with a higher PRAL score [7]. Yet, this finding could not be reproduced in a Swedish study of 911 elderly men with 115 cases through 18 years of follow-up [8]. Gender discrepancies have been suggested since a study of 1191 incident cases of T2D among ~ 65,000 Japanese showed an association with T2D in men only during 5 years of follow-up [9]. Finally, higher dietary acidity load was reported to associate with increased insulin resistance in a study of 1732 Japanese (> 90% men) [10].

A widely used approach to estimate dietary acidity load is the Potential Renal Acid Load (PRAL) score which is a validated proxy for renal net acid excretion [11]. Another estimation for the acidity of the diet is the NEAP score (Net Endogenous Acid Production). Both estimates seem to reflect a similar risk of incident diabetes [6, 7, 9, 10].

The PRAL score is based on dietary intake of protein and various micronutrients, phosphorus, potassium, calcium and magnesium, and takes into account the absorption rate of the nutrients in the gut, unlike the NEAP score, which only operates with potassium and protein intake [6]. A negative PRAL score reflects an alkalizing potential of the diet whereas a positive PRAL score reflects an acidifying potential of the diet.

The aim of the present study was to substantiate and elaborate previous findings and test if PRAL score associates with impaired glucose tolerance and incident diabetes in our study sample of middle-aged people from the general Danish population. Furthermore, in a cross sectional study of middle-aged individuals from the Danish general population, we aimed to test the hypothesis that a higher PRAL score associates with diabetes-related intermediary traits, including impaired beta-cell function and insulin resistance, derived from Oral Glucose Tolerance Tests (OGTT).

## Methods

The present study is based on two Danish population-based cohorts: the Diet, Cancer and Health cohort (DCH) and the Inter99 cohort (ClinicalTrials.gov ID no. NCT00289237).

### Diet, Cancer and health cohort

During 1993 to 1997, 160,725 Danish men and women were invited to participate in the DCH cohort; the inclusion criteria being age 50–64 years, living in the greater Copenhagen and Aarhus areas, born in Denmark and not registered with a previous cancer diagnosis in the Danish Cancer Registry. In total, 27,178 men and 29,875 women participated. However, 574 individuals were excluded due to cancer diagnosis before baseline, leaving 56,479 participants available for analysis.

The present study is based on data from 25,808 men and 28,843 women after exclusion of patients with known diabetes at baseline (*n* = 1371), participants with incomplete dietary registration (*n* = 53), participants with extreme values of self-reported energy intake (< 1000 KJ/day (*n* = 0) or > 20,000 KJ/day (*n* = 197)) and participants with missing values for BMI and lifestyle characteristics (diet, smoking and physical activity) (*n* = 207). Information on incident diabetes during the 15 years of follow-up was obtained from The National Diabetes Register [12] and dates of death were obtained from the Danish Civil Registration System. All other information was collected at baseline. A flowchart is given in Additional file 1: FigureS1.

In the DCH cohort, diet was monitored at recruitment by a 192-item FFQ that each participant received by mail before their visit to the study centre. The FFQ was designed specifically for this study population, aiming to capture the average intake of different food and beverage items over the past 12 months before study inclusion. Daily intakes of foods and nutrients were calculated for each participant by the software programme FoodCalc (www.ibt.ku.dk/jesper/foodcalc/). A description of the development and validation of this FFQ, and a detailed description of the estimation of the dietary intake of the population have been published [13–16]. Information on smoking habits and physical activity was obtained from questionnaires.

### Inter99 cohort

The Inter99 cohort is a non-pharmacological intervention study for the prevention of ischaemic heart disease [17]. Detailed description of the Inter99 study is given in the Additional file 1.

The present study is based on data from 2843 men and 2881 women after exclusion of participants with incomplete dietary registration (*n* = 150), participants with missing data from the OGTT (*n* = 359) and participants with extreme values of self-reported energy intake (< 1000 KJ/day (*n* = 6) or > 20,000 KJ/day (*n* = 93)). Additionally, 19 individuals had fasting serum C-peptide levels lower than 150 pmol/l and were, due to suspicion of type 1 diabetes, excluded from further analyses. Four hundred thirty-three individuals had missing information on smoking, physical activity, dietary intake or body mass index (BMI) and were thus excluded. A flowchart is given in Additional file 1: FigureS2.

Based on OGTT derived data, participants were characterised as having normal glucose tolerance (NGT) (*n* = 4288), impaired fasting glucose (IFG) (*n* = 474), impaired glucose tolerance (IGT) (*n* = 655) or screen detected, treatment-naive T2D (*n* = 214) according to the 1999 WHO criteria [18]. Additionally, a self-reported diabetes diagnosis was reported for 93 participants. In the present analytical protocol, individuals with combined IFG and IGT are presented together in the IGT group.

In the multivariate analyses of diabetes-related intermediary traits, participants with self-reported diabetes at baseline were excluded (*n* = 93) leaving 5631 participants eligible for analysis.

To assess beta cell function we used insulinogenic index and corrected insulin response as well as disposition index. To review insulin sensitivity we used Homeostatic Model Assessment of Insulin Resistance (HOMA-IR), Matsuda index of insulin sensitivity (ISI_Matsuda_) and BIGTT-Si. Calculations of these indices are given in Additional file 1.

The Inter99 study participants completed, at recruitment, a validated and self-administered food frequency questionnaire (FFQ) during their visit to the Research Centre [19]. They were asked to report their dietary intake during the month before examination. The FFQ included 198 questions on food items and beverages with additional questions regarding portion sizes of selected food items. All food items in the FFQ were linked to a food item in the Danish Food Composition Databank [20]. A detailed description of the questionnaire and estimation of the dietary intake of the population has been published [19]. Smoking status and physical activities were obtained from validated questionnaires as reported [17].

#### The potential renal acid load (PRAL) score

PRAL score was derived based on the estimated intake of several nutrients calculated from the FFQ used in Inter99 and DCH [6]:

$$ PRAL\ \left(\frac{mEq}{day}\right)=0.49\times protein\left(\frac{g}{day}\right)+0.037\times phosphorus\ \left(\frac{mg}{day}\right)-0.021\times potassium\ \left(\frac{mg}{day}\right)-0.013\times calcium\left(\frac{mg}{day}\right)-0.026\times magnesium\left(\frac{mg}{day}\right) $$

#### Statistical analyses

All statistical tests were performed using the R statistical package (http://cran.r-project.org/, version 3.1.3). A *p*-value of < 0.05 was considered statistically significant.

In the DCH cohort, Cox proportional hazards regression models with age as the time scale were used to estimate hazard ratios (HRs) of incident diabetes. Participants were censored at their date of death or emigration. PRAL score was analysed as categorised into quintiles, with the lowest category as the reference group. Two multivariate models were used: model 1 was adjusted for age (as time scale), smoking status (never-smoker/ex-smoker/current smoker), physical activity (metabolic equivalent of task (MET)) [21] and total fat, carbohydrate and energy intake, and model 2 was further adjusted for BMI. We tested the assumption of proportional hazards by visual inspection. Due to an uneven distribution of men and women across the quintiles and to address the previous diverging findings on men and women we conducted the analyses in combined datasets and stratified by sex.

In Inter99 logistic regression models were used to assess the association between PRAL score and IFG, IGT, and diabetes. Additionally, in Inter99 linear regression models were used to test whether PRAL score (as a continuous variable) was associated with diabetes-related intermediary traits in non-diabetic individuals only (*n* = 5631). Two multivariate models were used: model 1 was adjusted for age, sex, total fat, carbohydrate and energy intake, smoking status (never-smoker/ex-smoker/current smoker) and level of physical activity (0–2 h/week, 2–4 h/week, 4–7 h/week, 7–12 h/week) [22] and model 2 was further adjusted for BMI. To test whether the assumptions for linear regression analysis were fulfilled, all variables were inspected with regards to linearity as well as homogeneity of variance and normality of the residuals. Natural logarithmic transformation was used to approximate a normal distribution when needed. Test of effect modification was performed by introducing the interaction term in the linear models.

## Results

Baseline characteristics of included participants in the two cohorts are shown in Table 1 as stratified by quintiles of PRAL score. Individuals with a higher PRAL score had a higher intake of total and saturated fat, lower intake of fruits and vegetables and higher daily energy intake (see Table 1). In the subsequent analyses, we adjusted for these differences.

**Table 1:** Baseline characteristics of study participants in the Inter99 study and in the DCH cohort by quintiles of PRAL score

	Quintile 1	Quintile 2	Quintile 3	Quintile 4	Quintile 5	Total
Inter99 (*n* = 5631)						
Participants, n	1127	1126	1126	1126	1126	5631
PRAL range, mEq/d	−92; − 13	−13; − 3	−3; 4	4; 12	12; 90	−92; 90
Men, n (%)	469 (42)	530 (47)	487 (43)	581 (52)	722 (64)	2789 (50)
Age, years	50 (41; 55)	50 (40; 55)	45 (40; 50)	45 (40; 50)	45 (40; 50)	45 (40; 50)
BMI, kg/m^2^	26.2 ± 4.5	26.1 ± 4.4	26.1 ± 4.6	26.1 ± 4.3	26.3 ± 4.6	26.2 ± 4.5
Physical activity, min/week (%)						
0–112	117 (10)	136 (12)	133 (12)	157 (14)	155 (14)	698 (12)
142.5–225	246 (22)	248 (22)	261 (23)	256 (23)	256 (23)	1267 (23)
255–420	601 (53)	586 (52)	609 (54)	573 (51)	571 (51)	2940 (52)
450–720	163 (14)	156 (14)	123 (11)	140 (12)	144 (13)	726 (13)
Smoking, n (%)						
Current	539 (48)	444 (39)	420 (37)	390 (35)	396 (35)	2189 (39)
Former	283 (25)	304 (27)	292 (26)	287 (25)	267 (24)	1433 (25)
Never	305 (27)	378 (34)	414 (37)	449 (40)	463 (41)	2009 (36)
Energy intake, KJ/d	9286 ± 3082	9043 ± 2878	9049 ± 2861	9605 ± 2866	11,039 ± 3163	9604 ± 3064
Protein, g/d	76 ± 23	77 ± 23	79 ± 23	85 ± 23	103 ± 28	84 ± 26
Carbohydrates, g/d	293 ± 114	268 ± 96	263 ± 93	274 ± 92	302 ± 98	280 ± 100
Dietary fiber, g/d	23 ± 11	21 ± 8	20 ± 8	21 ± 8	23 ± 9	22 ± 9
Fat intake total, g/d	67 (51; 88)	72 (56; 94)	76 (59; 98)	84 (66; 107)	103 (79; 132)	80 (60; 105)
Saturated fat, g/d	25 (18; 35)	28 (21; 38)	29 (22; 39)	33 (25; 43)	40 (30; 53)	31 (22; 42)
Diet, Cancer and Health (*n* = 54,651)						
Participants, n	10,931	10,930	10,930	10,930	10,930	54,651
PRAL range, mEq/d	− 117; −10	−10; −3	−3; 4	4; 12	12; 89	−117; 89
Men, n (%)	3564 (33)	4215 (39)	4838 (44)	5789 (53)	7402 (68)	25,808 (47)
Age, years	55 (52; 60)	56 (52; 60)	56 (52; 60)	56 (52; 60)	55 (52; 60)	56 (52; 60)
BMI, kg/m^2^	25.6 ± 3.9	25.8 ± 4.0	26.0 ± 3.9	26.1 ± 4.0	26.4 ± 4.1	26.0 ± 4.0
Physical activity, MET-h/week	59 (39; 88)	56 (37; 84)	56 (36; 82)	56 (37; 83)	57 (37; 87)	57 (37; 85)
Smoking, n (%)						
Current	4551 (42)	4203 (38)	3723 (34)	3615 (33)	3605 (33)	15,713 (29)
Former	2963 (27)	2948 (27)	3125 (29)	3287 (30)	3390 (31)	19,697 (36)
Never	3417 (31)	3779 (35)	4082 (37)	4028 (37)	3935 (36)	19,241 (35)
Energy intake, KJ/d	9013 ± 2463	9026 ± 2405	9374 ± 2432	9948 ± 2467	11,384 ± 2745	9749 ± 2657
Protein, g/d	82 ± 23	86 ± 23	91 ± 23	99 ± 24	119 ± 29	95 ± 28
Carbohydrates, g/d	254 ± 78	240 ± 71	243 ± 70	252 ± 70	276 ± 75	253 ± 74
Dietary fiber, g/d	22 ± 8	20 ± 7	20 ± 7	20 ± 6	22 ± 7	21 ± 7
Fat intake total, g/d	67 (53; 83)	73 (58; 89)	78 (64; 96)	86 (70; 105)	104 (86; 126)	81 (64; 101)
Saturated fat, g/d	25 (19; 32)	28 (22; 35)	30 (24; 37)	33 (27; 41)	40 (33; 49)	31 (24; 39)
Food intake, g/d						
Red meat	63 (46; 84)	70 (52; 93)	77 (57; 101)	85 (63; 112)	105 (75; 141)	78 (56; 107)
Fish	32 (21; 46)	35 (23; 49)	37 (25; 52)	41 (28; 57)	49 (33; 70)	38 (25; 55)
Dairy products	263 (121; 515)	278 (138; 536)	289 (152; 547)	307 (172; 576)	344 (206; 617)	295 (156; 560)
Vegetables	180 (113; 272)	158 (100; 223)	151 (99; 213)	148 (97; 207)	155 (104; 213)	157 (102; 224)
Fruit	202 (107; 350)	154 (81; 254)	141 (74; 224)	131 (68; 204)	117 (61; 189)	145 (75; 240)

Data are mean ± standard deviation or median (inter quartile range) unless otherwise specified. Descriptive data on the Inter99 cohort are on non-diabetic individuals only. *MET* Metabolic Equivalent of Task, *PRAL* Potential Renal Acid Load

### Outcome from analyses of the DCH cohort

**Fig. 1:**
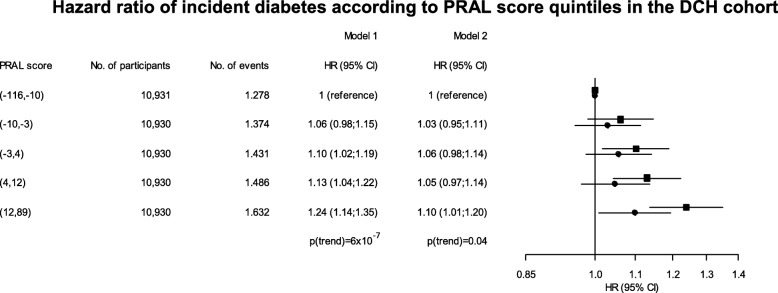
Hazard ratio (HR) of incident diabetes according to PRAL score quintiles in the full DCH cohort. Age is used as the time scale in the cox model. Model 1 is adjusted for fat, energy and carbohydrate intake, smoking and physical activity, while model 2 is adjusted for BMI, fat, energy and carbohydrate intake, smoking and physical activity. Squares indicate HR for model 1, and circles indicate HR for model 2. The *p*-value shown indicates significance of a trend test

**Fig. 2:**
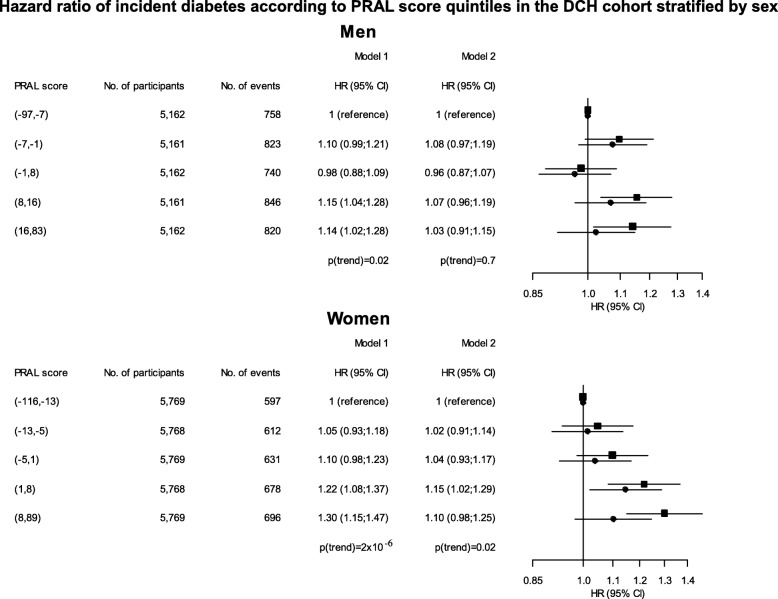
Hazard ratio (HR) of incident diabetes according to PRAL score quintiles in the DCH cohort stratified by sex. Age is used as the time scale in the cox model. Model 1 is adjusted for fat, energy and carbohydrate intake, smoking and physical activity, while model 2 is adjusted for BMI, fat, energy and carbohydrate intake, smoking and physical activity. Squares indicate HR for model 1, and circles indicate HR for model 2. The *p*-value shown indicates significance of a trend test

During a mean follow-up period of 15 years 7201 incident cases of diabetes occurred in the total study population. Multifactor adjusted HRs (model 1) of diabetes were 1.06 (95% CI 0.98; 1.15) (*p =* 0.12), 1.10 (95% CI 1.02; 1.19) (*p =* 0.02), 1.13 (95% CI 1.04; 1.22) (*p =* 0.003) and 1.24 (95% CI 1.14; 1.35) (*p =* 7 × 10^− 7^), respectively, for the second, third, fourth and fifth quintile versus the first quintile of PRAL score (*p*_trend_ = 6 × 10^− 7^) (Fig. 1). Further adjustment for BMI attenuated the HRs as HR for the 5th vs. the 1st quintile was 1.10 (95% CI 1.01; 1.20) (*p =* 0.03) (Fig. 1, model 2), but the same trend was observed across the quintiles (*p*_trend_ = 0.04). In the sex stratified analyses a similar association was observed for both men and women (Fig. 2). However, when BMI was included in the model the association with incident diabetes disappeared for men, but remained significant for women (*p*_trend_ = 0.02).

### Outcome from analyses of the Inter99 study

**Fig. 3:**
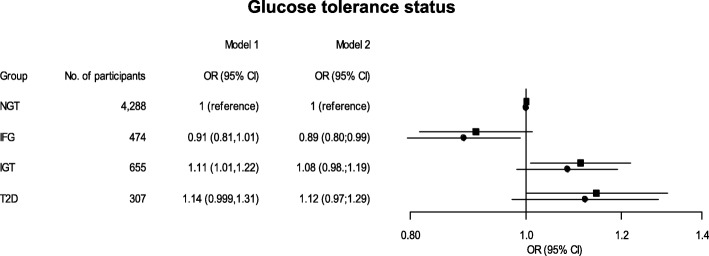
PRAL score and glucose tolerance status among participants of the Inter99 study. Odds ratios (OR) of impaired fasting glucose (IFG), impaired glucose tolerance (IGT) and type 2 diabetes (T2D) according to PRAL score as shown for one standard deviation of PRAL score in the total population (16.36 mEq/day). Model 1 is adjusted for age, sex, smoking, physical activity and fat, energy and carbohydrate intake, while model 2 is adjusted for age, sex, BMI, smoking, physical activity and fat, energy and carbohydrate intake. NGT = Normal Glucose Tolerance. Participants having combined IFG and IGT were classified as IGT. Squares indicate OR for model 1, and circles indicate OR for model 2

The multifactor-adjusted odds ratio (OR) for a one standard deviation higher PRAL score was 1.11 (95% CI 1.01; 1.22) (*p* = 0.03) for IGT (Fig. 3). The OR diminished with further adjustment for BMI (*p* = 0.11). No significant association was observed between PRAL score and T2D (Fig. 3) and PRAL score and IFG. However, when adjusting for BMI we found an association between lowered PRAL score and IFG with an OR of 0.89 (95% CI 0.80; 0.99) (*p* = 0.03).

A higher PRAL score was associated with lower OGTT-based measures of insulin resistance (model 1) as expressed by decreased BIGTT-*Si* (*p* = 4 × 10^− 7^) and ISI_Matsuda_ (*p* = 2 × 10^− 5^) as well as increased HOMA-IR (*p* = 0.001) (Table 2). Accordingly, a higher PRAL score was associated with higher serum insulin levels at fasting and 120 min during the OGTT (*p* = 2 × 10^− 4^ and *p* = 8 × 10^− 16^, respectively), and with higher plasma glucose levels at 120 min (*p* = 4 × 10^− 10^) (Table 2). No associations were observed between PRAL score and corrected insulin response (*p* = 0.2) or insulinogenic index (*p* = 0.3), both of which are indices of beta cell function. In addition, a higher PRAL score was associated with a lower OGTT-based disposition index (*p* = 0.004). All findings remained significant after further adjustment for BMI (model 2) and with similar effect sizes.

**Table 2:** Associations between PRAL score and diabetes-related intermediary traits in the baseline part of Inter99 study as well as means and medians for the individual quintiles

Variable	Quintile 1	Quintile 2	Quintile 3	Quintile 4	Quintile 5	Beta_Model 1_ (95%CI)	*P*_*Model 1*_	*P*_*Model 2*_
PRAL range (mEq/d)	−92; −13	−13; −3	−3; 4	4; 12	12; 90			
HBA1C (%)^a^	5.9 ± 0.5	5.8 ± 0.5	5.8 ± 0.5	5.8 ± 0.6	5.8 ± 0.5	−0.0009 (−0.002; −5 × 10^−5^)	0.04	0.01
Plasma glucose (mmol/l)								
Fasting^a^	5.6 ± 0.8	5.5 ± 0.8	5.5 ± 0.6	5.6 ± 0.9	5.5 ± 0.9	− 0.0003 (− 0.002; 0.001)	0.7	0.2
30 min^a^	8.7 ± 2.0	8.7 ± 1.9	8.6 ± 1.9	8.6 ± 1.8	8.7 ± 1.8	− 0.0009 (− 0.004; 0.002)	0.6	0.2
120 min	5.7 (4.8; 6.9)	5.9 (4.9; 6.9)	5.9 (4.9; 6.9)	6.0 (5.0; 7.0)	5.9 (5.0; 7.0)	0.2 (0.1; 0.2)	4 × 10^−10^	1 × 10^−8^
Serum insulin (pmol/l)								
Fasting	32 (22; 47)	33 (23; 50)	36 (24; 52)	35 (25; 53)	36 (25; 53)	0.2 (0.09; 0.3)	2 × 10^−4^	0.008
30 min	239 (171; 334)	234 (165; 346)	243 (181; 349)	257 (178; 376)	257 (183; 368)	0.07 (− 0.03; 0.2)	0.2	0.8
120 min	145 (86; 230)	151 (96; 246)	164 (103; 253)	162 (103; 273)	162 (95; 273)	0.6 (0.4; 0.7)	8 × 10^−16^	6 × 10^−14^
Measures of beta cell function								
Insulinogenic index	24.2 (16.5; 35.4)	23.4 (15.9; 34.7)	24.7 (17.2; 36.3)	25.8 (17.2; 38.8)	25.4 (17.2; 37.5)	0.06 (−0.06; 0.2)	0.3	0.8
Corrected insulin response	638 (392; 1051)	602 (392; 1033)	689 (413; 1107)	691 (410; 1146)	679 (413; 1094)	0.09 (−0.04; 0.2)	0.2	0.3
Measures of insulin sensitivity								
HOMA-IR	1.3 (0.89; 2.0)	1.3 (0.89; 2.1)	1.4 (0.94; 2.2)	1.4 (0.98; 2.2)	1.4 (0.97; 2.2)	0.2 (0.07; 0.3)	0.001	0.04
ISI_Matsuda_	8.4 (5.6; 11.7)	8.1 (5.3; 11.9)	7.8 (5.1; 11.1)	7.5 (5.1; 10.9)	7.6 (4.9; 10.8)	−0.2 (− 0.3; − 0.1)	2 × 10^−5^	0.001
BIGTT-Si^a^	9.7 ± 4.1	9.4 ± 4.1	9.3 ± 4.0	9.0 ± 4.0	8.8 ± 4.0	−0.02 (− 0.03; − 0.01)	4 × 10^−7^	4 × 10^−7^
Disposition Index	304 (211; 447)	291 (200; 410)	298 (203; 419)	297 (205; 412)	306 (206; 415)	−0.2 (− 0.3; − 0.05)	0.004	0.02

Data are from the Inter99 cohort (*n* = 5631) and are presented as mean ± standard deviation or median (inter quartile range) for each quintile of PRAL score as a description of the cohort. Effect sizes (95% confidence interval) are calculated using linear models with PRAL score as a continuous variable and are given as percentage increase for a one unit increase in PRAL score except for variables that have not been transformed by the natural logarithm in which case it is given as an increase per unit. The linear regression model 1 was adjusted for age, sex, smoking, physical activity and fat, energy and carbohydrate intake. The linear regression model 2 was further adjusted for BMI. BIGTT-Si was not adjusted for sex as this variable is included in the calculation of BIGTT-Si. Calculations of measures of beta cell function and insulin sensitivity were carried out as described in methods. Variables were transformed by the natural logarithm unless otherwise indicated by (a). Quintile values are raw data and are for descriptive purposes only

When testing for effect modification we introduced the interaction term PRAL×sex in the linear regression models, but the interaction terms were not significant (data not shown). However; to address diverging previous findings in men and women we further stratified by sex. We found that a higher PRAL score was associated with indices of insulin sensitivity in both women and men (Table 3), but the association with increased HOMA-IR with increased PRAL score was only apparent in women. Furthermore, an association between higher PRAL score and lower HbA1c was seen in men, but only after adjustment for BMI. No associations were found between PRAL score and indices of beta cell function in the sex stratified analyses, but a higher PRAL score was associated with lower disposition index in women (Table 3).

**Table 3:** Associations in the Inter99 study between PRAL score and diabetes-related intermediary traits stratified by sex as well as means and medians for the individual quintiles

Variable	Quintile 1	Quintile 2	Quintile 3	Quintile 4	Quintile 5	Beta_Model 1_ (95%CI)	*P*_*Model 1*_	*P*_*Model 2*_
Men	*n* = 558	*n* = 558	*n* = 557	*n* = 558	*n* = 558			
PRAL range (mEq/d)	− 92; −11	−11; −1	−1; 6	6; 15	15; 90			
HBA1C (%)^a^	6.0 ± 0.6	5.9 ± 0.6	5.9 ± 0.4	5.9 ± 0.7	5.9 ± 0.6	− 0.001 (− 0.003; 6 × 10^− 5^)	0.06	0.04
Plasma glucose (mmol/l)								
Fasting^a^	5.8 ± 1.0	5.7 ± 0.9	5.7 ± 0.7	5.7 ± 1.1	5.6 ± 0.9	− 0.001 (− 0.004; 0.0008)	0.2	0.1
30 min^a^	9.5 ± 2.1	9.1 ± 1.9	9.2 ± 1.8	9.2 ± 1.8	9.0 ± 1.8	−0.003 (− 0.007; 0.001)	0.2	0.09
120 min	5.6 (4.6; 7.0)	5.9 (4.9; 6.9)	5.9 (4.8; 6.9)	5.7 (4.8; 6.79)	5.9 (4.9; 7.0)	0.2 (0.1; 0.3)	3 × 10^− 6^	6 × 10^− 6^
Serum insulin (pmol/l)								
Fasting	34 (23; 52)	36 (24; 54)	38 (25; 56)	37 (25; 57)	37 (25; 55)	0.2 (0.02; 0.3)	0.02	0.052
30 min	244 (170; 356)	251 (165; 368)	255 (181; 380)	253 (173; 382)	264 (185; 396)	0.06 (−0.08; 0.2)	0.4	0.9
120 min	123 (67; 223)	141 (82; 233)	155 (83; 255)	143 (81; 242)	150 (89; 275)	0.6 (0.4; 0.8)	4 × 10^−9^	5 × 10^−9^
Measures of beta cell function								
Insulinogenic index	22.5 (15.0; 34.0)	23.5 (15.2; 34.9)	23.8 (16.2; 35.5)	23.1 (15.5; 36.3)	25.6 (16.6; 38.8)	0.08 (−0.09; 0.2)	0.3	0.6
Corrected insulin response	511 (306; 820)	557 (358; 932)	564 (353; 968)	563 (360; 891)	655 (384; 1008)	0.1 (−0.04; 0.3)	0.1	0.2
Measures of insulin sensitivity								
HOMA-IR	1.5 (0.9; 2.3)	1.5 (1.0; 2.3)	1.6 (1.0; 2.4)	1.6 (1.0; 2.4)	1.5 (1.0; 2.4)	0.1 (−0.02; 0.3)	0.09	0.2
ISI_Matsuda_	7.7 (4.9; 11.5)	7.3 (4.9; 11.1)	7.2 (4.8; 10.6)	7.1 (5.0; 11.0)	7.4 (4.6; 10.6)	−0.2 (−0.3; − 0.03)	0.02	0.04
BIGTT-Si^a^	8.3 ± 3.6	8.1 ± 3.5	8.0 ± 3.4	8.1 ± 3.5	8.1 ± 3.6	−0.02 (− 0.02; − 0.007)	3 × 10^−4^	3 × 10^−4^
Disposition Index	285 (188; 407)	273 (185; 390)	277 (188; 381)	282 (186; 382)	302 (197; 419)	−0.1 (− 0.3; 0.05)	0.2	0.3
Women	*n* = 569	*n* = 568	*n* = 568	*n* = 568	*n* = 569			
PRAL range (mEq/d)	−84; −15	−15; −5	−5; 1	1; 9	9; 77			
HBA1C (%)^a^	5.8 ± 0.8	5.7 ± 0.4	5.7 ± 0.4	5.7 ± 0.5	5.7 ± 0.5	−0.0002 (−0.001; 0.0009)	0.7	0.3
Plasma glucose (mmol/l)								
Fasting^a^	5.4 ± 0.7	5.3 ± 0.5	5.3 ± 0.5	5.3 ± 0.6	5.3 ± 0.9	0.001 (−0.0005; 0.003)	0.2	0.4
30 min^a^	8.1 ± 1.8	8.2 ± 1.7	8.1 ± 1.7	8.1 ± 1.8	8.2 ± 1.7	0.003 (− 0.002; 0.007)	0.3	0.8
120 min	5.7 (4.9; 6.7)	5.9 (4.9; 6.9)	6.0 (5.2; 6.9)	6.0 (5.1; 7.0)	6.0 (5.2; 7.1)	0.2 (0.08; 0.2)	3 × 10^−5^	7 × 10^−4^
Serum insulin (pmol/l)								
Fasting	31 (22; 46)	30 (22; 44)	34 (23; 51)	34 (24; 47)	33 (24; 50)	0.3 (0.1; 0.4)	8 × 10^−4^	0.03
30 min	238 (171; 325)	228 (168; 322)	238 (178; 324)	243 (179; 348)	253 (185; 347)	0.1 (−0.02; 0.3)	0.08	0.5
120 min	155 (104; 230)	162 (113; 246)	175 (114; 259)	178 (119; 277)	185 (123; 275)	0.5 (0.4; 0.7)	1 × 10^−8^	1 × 10^−6^
Measures of beta cell function								
Insulinogenic index	25.3 (17.6; 37.4)	24.4 (17.3; 34.4)	24.7 (17.8; 35.9)	26.0 (18.4; 39.5)	27.1 (18.6; 38.7)	0.07 (−0.09; 0.2)	0.4	0.8
Corrected insulin response	736 (483; 1224)	693 (454; 1131)	762 (456; 1223)	775 (494; 1278)	827 (488; 1352)	0.05 (−0.2; 0.3)	0.6	0.7
Measures of insulin sensitivity								
HOMA-IR	1.2 (0.84; 1.8)	1.2 (0.85; 1.8)	1.3 (0.89; 2.0)	1.3 (0.94; 1.9)	1.3 (0.91; 2.0)	0.3 (0.1; 0.4)	8 × 10^−4^	0.04
ISI_Matsuda_	8.8 (6.1; 12.1)	8.7 (6.0; 12.3)	8.2 (5.4; 11.7)	8.0 (5.7; 10.9)	7.9 (5.5; 11.0)	−0.3 (−0.5; −0.2)	2 × 10^−5^	0.002
BIGTT-Si^a^	10.7 ± 4.2	10.6 ± 4.1	10.3 ± 4.4	10.3 ± 4.1	10.0 ± 4.3	−0.03 (− 0.04; − 0.02)	8 × 10^−6^	8 × 10^−6^
Disposition Index	324 (225; 474)	316 (217; 434)	308 (204; 425)	312 (227; 431)	324 (223; 428)	−0.2 (− 0.4; − 0.08)	0.003	0.02

Data are from the Inter99 cohort and presented as mean ± standard deviation or median (inter quartile range) for each quintile of PRAL score as a description of the cohorts. Effect sizes (95% confidence interval) are calculated using linear models with PRAL score as a continuous variable and are given as percentage increase for a one unit increase in PRAL score except for variables that have not been transformed by the natural logarithm in which case it is given as an increase per unit. The linear regression model 1 was adjusted for age, smoking, physical activity and fat, energy and carbohydrate intake. The linear regression model 2 was adjusted for age, BMI, smoking, physical activity and fat, energy and carbohydrate intake except for BIGTT-Si, which was not adjusted for BMI (as this variable is included in the calculation of BIGTT-Si). Calculations of measures of beta cell function and insulin sensitivity were carried out as described in methods. Variables were transformed by the natural logarithm unless otherwise indicated by (a). Quintile values are raw data and are for descriptive purposes only

### Sensitivity analysis

In the Inter99 cohort we further did a sensitivity analysis where we adjusted for family history of diabetes, hypertension and dietary patterns (see Additional file 1: TableS1). This information was not available to us in the DCH cohort, and so the sensitivity analysis was only conducted in the Inter99 cohort. The sensitivity analysis showed comparable associations as demonstrated in the primary analyses except the associations between PRAL score and HbA1c and disposition index. In the sensitivity analysis the two latter associations became non-significant (see Additional file 1: TableS1).

## Discussion

In the DCH cohort of more than 54,000 individuals with ~ 7200 incident cases of diabetes after 15 years of follow-up, we demonstrated a positive relationship between PRAL score and development of incident diabetes, substantiating previous findings [6–10]. Moreover, in baseline data from the Inter99 study of 5631 non-diabetic individuals with OGTT-derived data, we found that a higher PRAL score was associated with reduced insulin sensitivity, but not with changes in proxies of beta cell function. In addition, higher PRAL score was associated with a lower OGTT-based disposition index. We did not observe any major diverging results between the sexes, and so we cannot confirm that any specific differences exist in regards to PRAL score and sex. When adjusting for BMI in the DCH cohort, only the increased HR between the first and the fifth quintiles remained significant. Furthermore, when adjusting for BMI, the significant trend seen in males disappeared, but remained significant in women. In the Inter99 cohort, adjustment for BMI had no effect on the analyses. Since the individuals with a higher PRAL score generally had a higher overall energy intake, the increased BMI may be part of the explanation for the increased incidence of diabetes. However, since the associations persist even after adjustment for BMI, it shows that there is indeed an association between PRAL score and incident diabetes as well as diabetes intermediary traits which is independent of BMI.

Despite a lack of association with indices of beta-cell function in the Inter99 study, we did find an association with lower disposition index as an indication of a decreased beta cell secretion of insulin at the concomitant level of insulin resistance. Hence, we cannot completely discard an association between PRAL score and beta-cell function. Higher PRAL score is consistent with a generally unhealthy diet, which is reflected in our finding that individuals with a higher PRAL score also had a higher intake of total and saturated fat, lower intake of fruits and vegetables and higher daily energy intake. We have accounted for these confounders by adjusting for dietary fat, carbohydrate and total energy intake in our analyses.

The different nutrients, especially protein, embedded in the calculation of the PRAL score may come from various sources. Since the amino acid composition of plant proteins is different from the composition of animal proteins, it is possible that ingestion of plant proteins will have a different effect on the tissue acidity than animal proteins. Indeed, a previous study showed an association between incident T2D and animal protein intake, but not with plant protein intake [23]. One could therefore speculate that a type of measure where proteins were reported as of their origin or corresponding amino acids would provide useful information about the underlying theories of dietary acidity load and diabetes.

The amount of dietary fiber ingested are surprisingly similar across the quintiles. However, the food items from where the dietary fiber is obtained may vary considerably and may therefore have different impacts on the PRAL score. For instance, dietary fibers originating from vegetables and fruits will have an alkalizing effect and thus decrease the PRAL score, whereas dietary fiber originating from whole grain may have a different effect on the PRAL score [1]. As appears from the baseline table (Table 1) of the DCH cohort, the dietary fiber ingested in the lower quintiles must come from fruits and vegetables, whereas the dietary fiber in the higher quintiles originate from other food items, although we cannot say for certain which ones. Additionally, it is noteworthy that the quintiles with the highest PRAL scores are also the ones with the highest number of current smokers. This is the case in both the DCH cohort and the Inter99 cohort. It cannot be excluded that smoking status might influence food preferences. Indeed, a study by Endoh et al. shows, that the dietary patterns in Japanese men and women differ between smokers and non-smokers [24].

An intervention study conducted in American adults showed that individuals would be able to lower their PRAL score by 13 units when following a vegan diet for two to three days a week, while a reduction of almost 30 units was seen in individuals following the vegan diet every day in a week [25]. If we examine our data from the DCH cohort, we find that a reduction of 30 units would be sufficient to move an individual from Q4 to Q1. With a reduction of just 12 units an individual would step down an entire quintile. Despite the modest increased risk of diabetes between the lowest and the highest quintile, diabetes is still major burden in society today, and even slight reductions in this risk through a diet change might have considerable impact on public health.

Our study has limitations. First, in the present epidemiological studies, we did not use the hyperinsulinemic euglycemic clamp to measure insulin sensitivity, as this would not be feasible in such large cohorts; still the surrogate measures applied in the current study, HOMA-IR, ISI_Matsuda_ and BIGTT-Si, have all been validated in previous studies and provide physiologically relevant proxies for insulin sensitivity [26–28]. Additionally, to overcome any uncertainties in the individual surrogate measures, we have used several different proxies of insulin sensitivity to measure the same outcome. Second, and most important, in our study of incident diabetes, information was collected at baseline only. Consequently, all conclusions based on the DCH cohort rely on the assumption that the study participants have not changed their diet or lifestyle substantially over the following 15 years of follow-up. Third, the diagnosis of diabetes was based on The Danish National Diabetes Register which does not clearly distinguish between patients with type 1 diabetes or T2D [12]. Yet, individuals in our study were at least 50 years of age at baseline, and since newly diagnosed type 1 diabetes is quite rare in Denmark above this age we do not expect this lack of diagnostic accuracy to bias our analytical outcomes. The PRAL score in our two cohorts ranged from around − 100 to 100 and with a median range of − 20.6 to 18.6 in the Inter99 cohort and − 16.3 to 18.0 in the DCH cohort, respectively. These median ranges are roughly the same as in the two studies by Kiefte-de Jong et al. (− 17.4 to 21.3) and Fagherazzi et al. (− 23.0 to 14.3). However, the PRAL score range in the study by Xu et al. was from − 40 to 323. For histograms of the PRAL scores in the two cohorts, please see Additional file 1: FigureS3 and FigureS4. We believe that these between study differences may be related to differences in dietary patterns and the demographics of the study populations. Furthermore, the variations in PRAL score may occur due to differences in how the questionnaires and food composition tables are structured between studies.

## Conclusions

We confirm the association of a high acidity load with incident T2D and suggest that the risk of T2D might be mediated partially through a decrease in insulin sensitivity. Although the present study and previous reports suggest a link between dietary acidity load and incident diabetes among middle-aged people, carefully designed and conducted dietary interventions are needed to elucidate whether a causal link exists between the two and which mechanisms might be involved.

## Additional files

Additional file 1: Supplements. (DOCX 85 kb)

## Abbreviations

BIGTT: Beta-cell function; Insulin sensitivity; Glucose; Tolerance; Test
BIGTT-Si: An OGTT-derived index of insulin sensitivity (Si)
DCH: The Diet, Cancer and Health cohort
FFQ: Food frequency questionnaire
IFG: Impaired fasting glucose
IGT: Impaired glucose tolerance
ISI_Matsuda_: Matsuda index of insulin sensitivity
MET: Metabolic equivalent of task
NEAP: Net Endogenous Acid Production
OGTT: Oral glucose tolerance test
PRAL: Potential Renal Acid Load
T2D: Type 2 diabetes
