# Supplementary material for: Population-based studies of relationships between dietary acidity load, insulin resistance and incident diabetes in Danes
Source: Nutr J. 2018 Oct 6;17:91. doi: 10.1186/s12937-018-0395-1 (PMC6173920; doi:10.1186/s12937-018-0395-1)
Supplement: Supplementary file 1 — Supplements. (DOCX 85 kb) [file 12937_2018_395_MOESM1_ESM.docx]

**Additional file 1**

Supplementary methods showing the calculations and explaining the different indices used in the article as well as explaining in detail the Inter99 study and how measurements of anthropometrics and the oral glucose tolerance test were carried out.

**Calculation of indices of beta cell function**Both insulinogenic index and corrected insulin response are surrogate measures of oral glucose stimulated insulin secretion [1] and are calculated as:

Insulinogenic index:
(Serum insulin at 30 min – fasting serum insulin) / (plasma glucose at 30 min).

Corrected Insulin Response:
100 x serum insulin at 30 min /(plasma glucose at 30 min × (plasma glucose at 30 min – 3.89)).

Disposition Index is a surrogate measure of beta cell secretion of insulin at the concomitant level of insulin resistance and is calculated as:

Insulinogenic index × ISI_Matsuda_.

**Calculation of indices of whole-body insulin sensitivity**Homeostatic Model Assessment of Insulin Resistance (HOMA-IR) was applied to assess the insulin sensitivity at the fasting state, and was calculated as:
(Fasting insulin x fasting plasma glucose)/135 [2]

Matsuda index of insulin sensitivity (ISI_Matsuda_) was calculated as:

$$\frac{1000}{\sqrt{G0 x I0 x G\left( mean \right)x I(mean)}}$$

Where G0 is fasting plasma glucose, I0 is fasting plasma insulin, G(mean) is mean glucose concentration during the OGTT and I(mean) is the mean insulin concentration during the OGTT [3].

BIGTT-Si [4] was calculated as:

exp[4.90-(0.00402×I_0_) - (0.000556×I_30_) -(0.00127×I_120_) - (0.152×G_0_) - (0.00871×G_30_) -

(0.0373×G_120_) - (0.145×sex) - (0.0376×BMI)]

**Sensitivity analysis**

We performed a sensitivity analysis in the Inter99 cohort in addition to the main analysis where we, apart from the adjustments in model 1, further adjusted for family history of diabetes (yes/no), dietary patterns (healthy/medium healthy/unhealthy) and hypertension (yes/no). Family history of diabetes was defined as having one or both parents with diabetes and hypertension was defined as: systolic blood pressure > 140 mmHg and/or diastolic blood pressure > 90 mmHg and/or hypertension treatment.
The sensitivity analysis did not change the overall results of the primary analyses apart from the associations between PRAL score and HbA1c and disposition index, which became non-significant (see TableS1).

**The Inter99 Study**

A random sample of 13,016 individuals living in Copenhagen County from seven different age groups (all 40-50 years) was drawn from the Civil Registration System and invited for the study, and a total of 6,784 (52%) attended the baseline examination, which took place from March 1999 to January 2001.
Information on lifestyle, education, working conditions, chronic diseases, use of health care system and psychosocial factors were obtained using questionnaires.
Participants had their blood pressure measured twice using a mercury sphygmomanometer after five minutes of rest. Height without shoes was measured to the nearest cm. Weight without shoes and overcoat was measured to the nearest kg and BMI was calculated. Waist and hip circumference was measured in cm and waist/hip ratio calculated.[5]

Participants without known diabetes underwent, after an overnight fast, a 75 g OGTT with plasma glucose and serum insulin measured at fasting, 30 and 120 min after the oral glucose load. Glucose was analysed by hexokinase 1G6P-DH (Boehringer Mannheim) [6] and fasting serum insulin was measured by Elisa excluding des(31,32) and intact proinsulin (Dako Diagnostics Ltd., Ely, UK) [5-7]. HbA1c was taken in a capillary tube and analyzed by the principles of an ion-exchange high-performance liquid chromatography Bio-Rad variant.

**Table S1 Sensitivity analysis**

| Variable | *P_Model 1_* | *Sensitivity* |
| --- | --- | --- |
| PRAL range (mEq/d) |  |  |
| HBA1C (%)^a^ | 0.04 | 0.3 |
| Plasma glucose (mmol/l)  Fasting^a^  30 min^a^  120 min | 0.7  0.6  4×10^-10^ | 0.5  0.2  6×10^-5^ |
| Serum insulin (pmol/l)  Fasting  30 min  120 min | 2×10^-4^  0.2  8×10^-16^ | 0.01  0.7  2×10^-7^ |
| Measures of beta cell function  Insulinogenic index  Corrected insulin response | 0.3  0.2 | 0.6  0.3 |
| Measures of insulin sensitivity  HOMA-IR  ISI_Matsuda_  BIGTT-Si^a^  Disposition Index | 0.001  2×10^-5^  4×10^-7^  0.004 | 0.04  0.01  3×10^-7^  0.051 |

Data are from the Inter99 cohort (n=5,631). p-values are calculated using linear models with PRAL score as a continuous variable. The linear regression model 1 was adjusted for age, sex, smoking, physical activity and fat, energy and carbohydrate intake, as in the main text. In the sensitivity analysis, we further adjusted for hypertension, family history of diabetes and dietary patterns. BIGTT-Si was not adjusted for sex as this variable is included in the calculation of BIGTT-Si. Variables were transformed by the natural logarithm unless otherwise indicated by (a).

**Figure S1 Flowchart of the Diet, Cancer and Health cohort**

Diet, cancer and health

n=56,479

Diet, cancer and health

n=54,651

Known diabetes

n=1,371

Incomplete dietary registration

n=53

Extreme values of self reported energy intake

n=197

Missing values for BMI, energy, carbohydrate or fat intake, smoking or physical activity

n=207

**Figure S2 Flowchart of the Inter99 cohort**

Inter99

n=6,784

Inter99

n=5,724

Incomplete dietary registration

n=150

Extreme values of self- reported energy intake

n=99

Missing values for BMI, energy, carbohydrate or fat intake, smoking or physical activity

n=433

Missing OGTT data

n=359

Fasting serum C-peptide<150pmol/l

n=19

**Figure S3 Histogram showing the distribution of PRAL score in the DCH cohort**
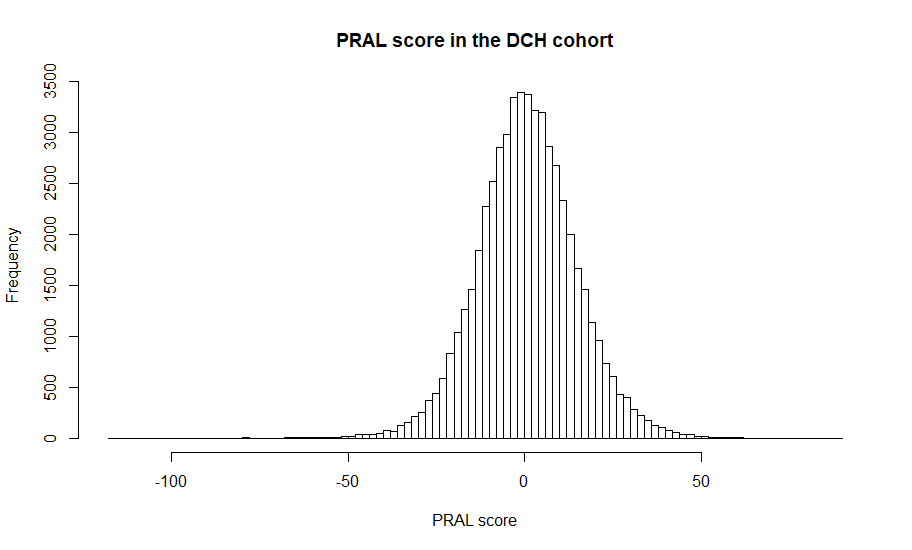


**Figure S4 Histogram showing the distribution of PRAL score in the Inter99 cohort**
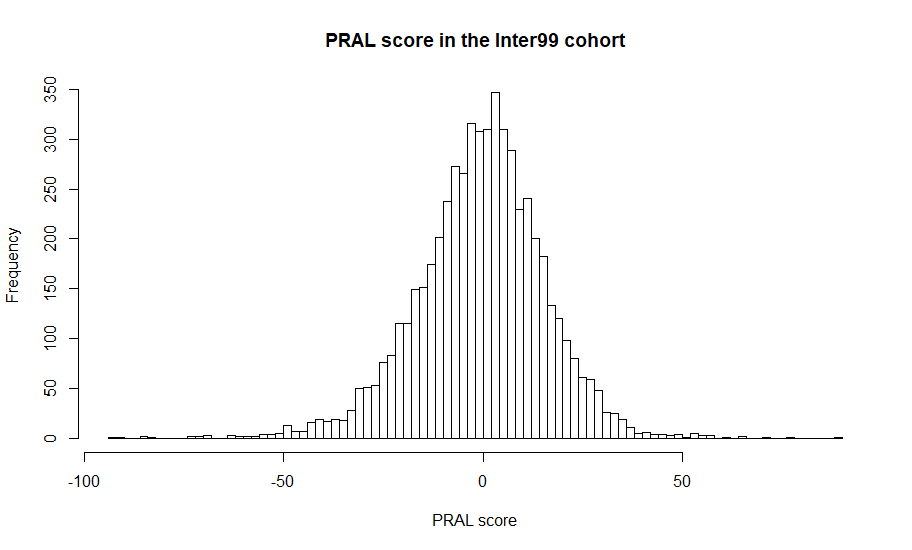


**References**

1. Hanson RL, Pratley RE, Bogardus C, Narayan KM, Roumain JM, Imperatore G, et al. Evaluation of simple indices of insulin sensitivity and insulin secretion for use in epidemiologic studies. Am J Epidemiol. 2000;151:190-198.

2. Matthews DR, Hosker JP, Rudenski AS, Naylor BA, Treacher DF, Turner RC. Homeostasis model assessment: insulin resistance and beta-cell function from fasting plasma glucose and insulin concentrations in man. Diabetologia. 1985;28:412-419.

3. Gutch M, Kumar S, Razi SM, Gupta KK, Gupta A. Assessment of insulin sensitivity/resistance. Indian J Endocrinol Metab. 2015;19:160-164.

4. Hansen T, Drivsholm T, Urhammer SA, Palacios RT, Volund A, Borch-Johnsen K, et al. The BIGTT test: a novel test for simultaneous measurement of pancreatic beta-cell function, insulin sensitivity, and glucose tolerance. Diabetes Care. 2007;30:257-262.

5. Jorgensen T, Borch-Johnsen K, Thomsen TF, Ibsen H, Glumer C, Pisinger C. A randomized non-pharmacological intervention study for prevention of ischaemic heart disease: baseline results Inter99. Eur J Cardiovasc Prev Rehabil. 2003;10:377-386.

6. Glumer C, Jorgensen T, Borch-Johnsen K. Prevalences of diabetes and impaired glucose regulation in a Danish population: the Inter99 study. Diabetes Care. 2003;26:2335-2340.

7. Lau C, Faerch K, Glumer C, Tetens I, Pedersen O, Carstensen B, et al. Dietary glycemic index, glycemic load, fiber, simple sugars, and insulin resistance: the Inter99 study. Diabetes Care. 2005;28:1397-1403.
